# Hyde on the Skin

**Published:** 1900-03

**Authors:** 


HYDE ON THE SKIN. New (5th) edition. A Practical Treatise on Diseases of the Skin, for the use of Students and Practitioners. By J. Nevins Hyde, A.M., M.D., Professor of Dermatology and Venereal Diseases in Rush Medical College, Chicago. In one octavo volume of 866 pages, with 111 engravings and 24 full-page plates, 8 of which are colored. Cloth, $4.50 net. Leather, $5.50 net.

The fifth edition of this deservedly popular work shows several new subjects and the rearrangement of much of the old text. Other parts should have been brought more up to date, to match. It would have been agreeable to have seen the frontispiece transposed with one of the other plates. The definitions of adjectives in the introductory parts are very useful. Absorption, as credited to the skin, is doubtful in the light of the latest experiments. Chapter on general therapeutics excellent, but it is to be wished that agents and remedies could have been put in alphabetic order. Classification that of present American acceptance. The suggestion that a sebaceous tumor should be emptied before excision is not good surgery. Remarks on the evil effects of "blood purifiers," the indiscriminate use of arsenic, and the belief in danger of "driving in" an eruption are most sensible. The beneficial effects of thyroid extract in psoriasis not mentioned in the chapter on that disease. Colored plates very good. The black and white cuts medium to bad. Milliamperemeter he considers not essential in electrolytic treatment. No book is perfect, this is one of the best.

